# The concentration of potentially toxic elements (PTEs) in drinking water from Shiraz, Iran: a health risk assessment of samples

**DOI:** 10.1007/s11356-022-23535-2

**Published:** 2022-11-02

**Authors:** Amin Mohammadpour, Zahra Emadi‬, Mohammad Reza Samaei, Khaiwal Ravindra, Seyedeh Masoumeh Hosseini, Mohammad Amin, Mojtaba Samiei, Leili Mohammadi, Razyeh Khaksefidi, Amin allah Zarei, Mohadeseh Motamed-Jahromi, Amin Mousavi Khaneghah

**Affiliations:** 1 Department of Environmental Health Engineering, School of Public Health, Shiraz University of Medical Sciences, Shiraz, Iran; 2 Department of Environmental Health Engineering, School of Public Health, Shahrekord University of Medical Sciences, Shahrekord, Iran; 3 Department of Community Medicine & School of Public Health, Post Graduate Institute of Medical Education & Research (PGIMER), Chandigarh, 160012 India; 4 Department of Public Health and Food Hygiene, School of Veterinary Medicine, Shiraz University, PO Box 1731, Shiraz, Postal code 71345 Iran; 5 Department of Mechanical Engineering, Shiraz University, Shiraz, Iran; 6 Environmental Health, Infectious Diseases and Tropical Medicine Research Center, Zahedan University of Medical Sciences, Zahedan, 9816743463 Iran; 7 Health Sciences Research Center, Torbat Heydariyeh University of Medical Sciences, Torbat Heydariyeh, Iran; 8 Department of Medical-Surgical Nursing, Nursing School, Fasa University of Medical Sciences, Fasa, Iran; 9 Department of Fruit and Vegetable Product Technology, Prof. Wacław Dąbrowski Institute of Agricultural and Food Biotechnology – State Research Institute, 36 Rakowiecka St, 02-532 Warsaw, Poland

**Keywords:** Drinking water, PTEs, Pollution index, Degree of contamination, Correlation analysis, Health risk assessment, Monte Carlo simulation, Sensitivity analysis

## Abstract

The existence of potentially toxic elements (PTEs) in water bodies has posed a menace to human health. Thus, water resources should be protected from PTEs, and their effect on the exposed population should be investigated. In the present investigation, the concentrations of PTEs such as lead (Pb), mercury (Hg), manganese (Mn), and iron(Fe) in the drinking water of Shiraz, Iran, were determined for the first time. In addition, hazard quotient, hazard index, cancer risk, and sensitivity analysis were applied to estimate the noncarcinogenic and carcinogenic impacts of Pb, Hg, Mn, and Fe on exposed children and adults through ingestion. The mean concentrations (µg/L) of Pb, Hg, Mn, and Fe were 0.36, 0.32, 2.28, and 8.72, respectively, in winter and 0.50, 0.20, 0.55, and 10.36, respectively, in summer. The results displayed that Fe concentration was more than the other PTEs. PTE concentrations were lower than the standard values of the Environment Protection Agency and World Health Organization. Values of the degree of contamination and heavy metal pollution index for lead, mercury, manganese, and iron were significantly low (< 1) and excellent (< 50), respectively. Based on the Spearman rank correlation analysis, positive and negative relationships were observed in the present study. The observations of the health risk assessment demonstrated that mercury, lead, iron, and manganese had an acceptable level of noncarcinogenic harmful health risk in exposed children and adults (hazard quotients < 1 and hazard index < 1). The carcinogenic risk of lead was low (< E − 06), which can be neglected. Monte Carlo simulation showed that water intake rate and mercury concentration were the most critical parameters in the hazard index for children and adults. Lead concentration was also the most crucial factor in the cancer risk analysis. The results of the present study proved that the drinking water of Shiraz is safe and healthy and can be confidently consumed by people.

## Introduction

Food products and water perform an indispensable and essential role in the life of humans, animals, plants, and other living organisms. Hence, their quality can affect our daily life (Amiri et al. 2021). Therefore, the required water should not contain undesired contaminations, microorganisms, and harmful chemicals (Lanjwani et al. 2020; Quan et al. 2021; Wang et al. 2021). However, unfortunately, the water resources have been facing a severe crisis because of the express and disordered population growth, irresponsible administration, and industrial-agricultural actions (RadFard et al. 2019; Saleh et al. 2019; Ashoori et al. 2022). Groundwater and surface water are significant resources of water supply systems. Water resources are treated and distributed in tap water and bottled mineral water. Thus, they can be consumed as drinking water (Ab Razak et al. 2015).

In Iran, people generally use tap water as the main source of drinking water. Water is posed as a passive carrier for the transport of different pollutants, which causes numerous health complications for humans. It should be pointed out that poor-quality drinking water results in 80% of diseases (Qasemi et al. 2018). Thus, accessibility to safe and uncontaminated water is urgent in a healthy lifestyle (Mirzabeygi et al. 2017). However, drinking water contamination may occur by natural and anthropogenic activities. Rock weathering, soil erosion, and ore deposits are the most remarkable natural sources of contaminants in drinking water. In addition, wastewater, agricultural, industrial, and mining activities are anthropogenic sources of contaminants. Among different contaminants that can affect the quality of water resources, PTEs are the most important types (Khan et al. 2015).

PTEs include copper (Cu), arsenic (As), iron (Fe), molybdenum (Mo), cobalt (Co), zinc (Zn), mercury (Hg), lead (Pb), and manganese (Mn) (Bounar et al. 2020; Heshmati et al. 2020). The effects of PTEs are related to their concentrations (Hu et al. 2022; Luo et al. 2022; Rezaei et al. 2020). Based on the reports, low concentrations of PTEs can act as nutrients and cofactors in enzymatic metabolism. However, high concentrations can be toxic and have prohibitive/deadly effects on humans, animals, plants, and microorganisms (Fallahzadeh et al. 2017; Mishra et al. 2019). Bioaccumulation and poor biodegradability are the most significant adverse effects of PTEs, which can cause damage to human health (Mohammadi et al. 2019). PTEs accumulate in body organs like the liver, brain, kidneys, and bones for decades and years. United States Agency for Toxic Substances and Disease Registry recognized Pb, As, and Hg as priority contaminants (Kamunda et al. 2016).

Humans can be exposed to Hg through several sources like ingestion by drinking water, ambient air, vaccine, fish, and occupational and home exposure (thermostats, fluorescent light bulbs, tattoo dye, batteries, lightening creams for skin, fluid of contact lens, and dental amalgams). Approximately 80 and 100% of Hg are estimated to be absorbed by vapor and oral consumption, respectively. The solubility of Hg in lipids is high and can easily enter the cells. In addition, Hg can be distributed in the brain and kidneys and easily transferred to the fetus through the placenta (Hyman 2004; Zoghi et al. 2022). Galena mineral (PbS) exists in the natural composition of the earth's crust. Pb also exists in smoking, pesticides, automobile emissions, mining, and paint (Basheer et al. 2020). Pb is a nonvital, highly toxic, carcinogenic element in the aquatic environment (Gao et al. 2022). Lead causes oxidative stress and hurts the organs like the brain, kidneys, cells, and nerves, particularly in pregnant women and children. It also has other chronic effects like abdominal pain, headache, lung and stomach cancers, and blood pressure (Hernández et al. 2020; Muhammad et al. 2011).

The quantity of Mn and Fe in water depends on the geological characteristics of the area (Grazuleviciene et al. 2009). Fe enters into the water bodies through different processes like effluents of iron and steel industries and seepage water from iron rocks and minerals (Haldar et al. 2020). The presence of Fe in the body has been recognized as a vital factor for the health of humans. It is present in the structure of enzymes, hemoglobin, and protein. It can catalyze the metabolisms in the body. Nevertheless, it has an endurable upper intake rate in adults (about 45 mg/day) (Basheer et al. 2020). Accumulation of excessive Fe has predominantly occurred in the liver, heart, pancreas, skin, pituitary, and joints after the age of 40, which can cause fibrosis of the liver, cirrhosis, cardiac diseases, diabetes, hyperpigmentation, hypogonadotropic hypogonadism, and arthritis (Papanikolaou and Pantopoulos 2005). Mn is consumed in ceramic and glass industries, batteries, and gasoline anti-knock additive production. Mn is a portion of enzymes that can catalyze fat and protein metabolisms and is also effective in digestion, growth of bone, metabolism of carbohydrates, production of energy for cells, and blood sugar regulation (Taylor et al. 2006). It should be stated that high concentrations of Mn lead to a reduction in fetal weight and can cause retardation in the improvement of internal and skeleton organs. Also, Mn has other hazardous effects like aberrations in chromosomes and damage to DNA in the fetus (Grazuleviciene et al. 2009). Their toxic and nontoxic effects should be investigated based on the mentioned effects of PTEs on the human body and health.

The evaluation of PTE exposure among the occupants in a particular area is carried out by health risk assessment (HRA) (Mohammadi et al. 2019). HRA can also estimate the hazards to human health and the level of hazard risk (Soleimani et al. 2022). HRA determines the noncarcinogenic and carcinogenic impacts of different compounds (Qu et al. 2012). Cancer is posed as a critical world health concern. Thus, proving the scientific relationship between water quality and cancer is valid and significant (Burton and Cornhill 1977). The primary purposes of the present research were to consider the PTEs concentrations (Pb, Hg, Mn, and Fe) in the drinking water of Shiraz city and risk assessment of PTEs for children and adults. PTE concentrations in groundwater (wells) of Shiraz have been investigated in various research. However, the present research only determined PTEs concentrations in Shiraz’s potable water (tap water).

## Materials and methods

### Study area

The study area (Shiraz) is situated in the central part of Fars Province (southwest of Iran). Shiraz lies between latitudes 29° 36′ 36″ N and longitudes 52° 32′ 33″ E at an elevation of 1585 m and a surface area of 240 km^2^. The climate of Shiraz is hot and semi-arid, with a population density of 6522 people per km^2^. Also, geological characteristics of Shiraz demonstrate the presence of carbonates (limestone) and siliciclastic (shale and sandstone) parent rock of Razak, Asmari, and Jahrom formations of Paleogene/Neogene deposits (Habibi et al. 2018). A global position system (GPS) recorded the characteristics in sampling sites.

### Sample collection and preparation

**Fig. 1:**
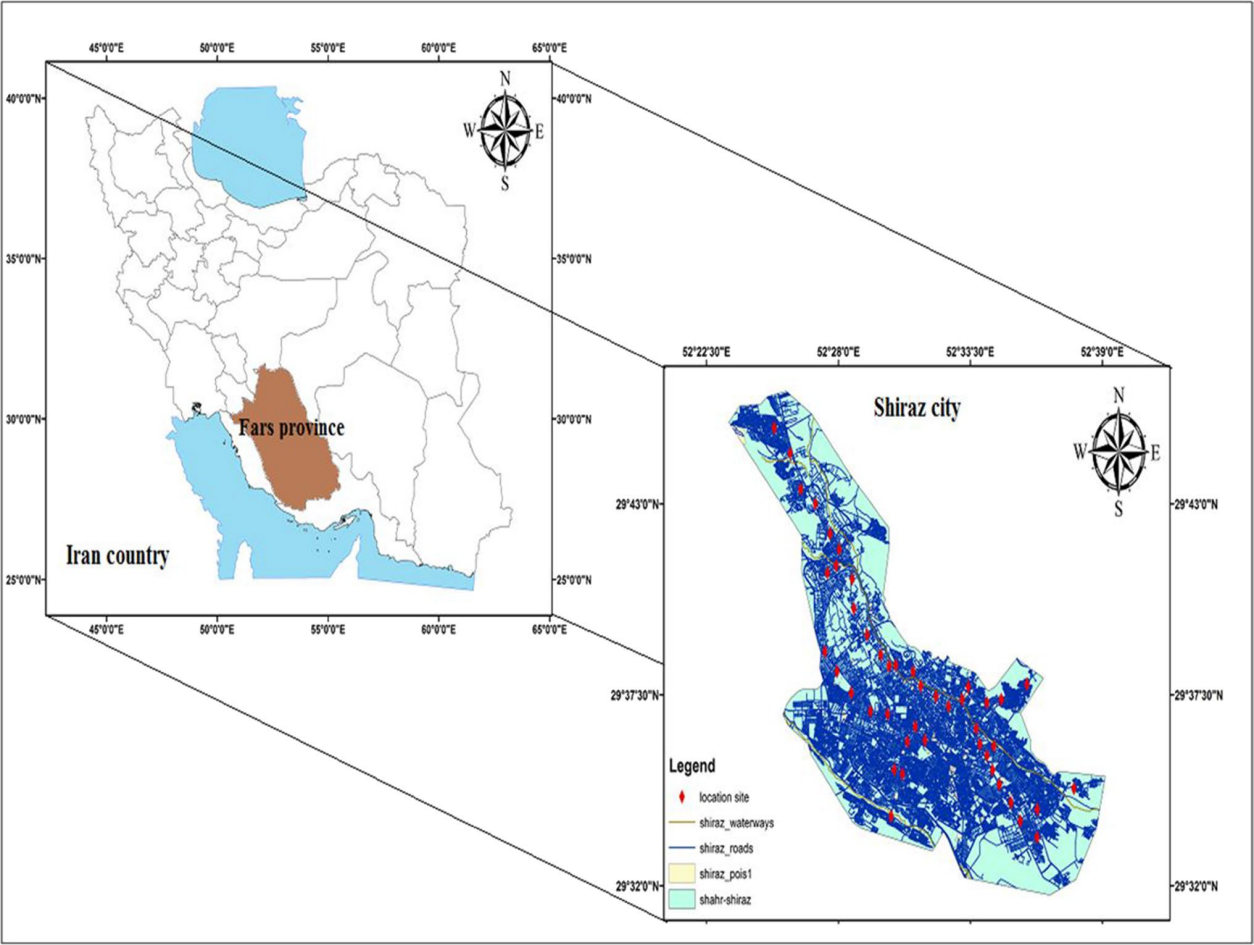
Location map of sampling

In the winter and summer of 2021, 90 samples were collected from Shiraz’s drinking water (tap water at homes without home water treatment systems) (Fig. 1). It should be noted that 45 samples in winter and 45 in summer were collected. Polyethylene bottles were washed before sampling (with deionized water). After sampling, concentrated HNO_3_ solution (2 mL) was added to water samples (pH < 2) (for the stabilization of PTEs) (Mohod and Dhote 2013; Nour and El-Sorogy 2020). Then, all samples were conveyed on ice to the laboratory and stored in a refrigerator (< 4 °C). In addition, all of the reagents were of analytical grade.

### Analysis

The collected acidified samples were filtered (Whatman Grade. 2). Then, the acidified/filtered water samples were analyzed for determination of PTEs (Pb, Hg, Mn, and Fe) based on the Standard Method for Examination of Water and Wastewater (APHA 2005) by inductively coupled plasma-mass spectrometry (ICP-MS) (Agilent 7500, USA). Also, the adverse impact of PTEs is evaluated by the degree of contamination (C_d_) (Eqs. 1 and 2).

1 $${C}_{d} = \sum\nolimits_{i =1}^{n}{Cf}_{i}$$

2 $${Cf}_{i }= \frac{{M}_{i}}{{S}_{i}} -1$$

*Cf*_*i*_, *M*_*i*_, and *S*_*i*_ are the contamination factor of PTEs, measured PTEs, and standard values of PTEs, respectively. Based on C_d_, water quality is categorized into C_d_ > 3 (high contamination), 1 < C_d_ < 3 (moderate contamination), and C_d_ < 1 (significantly low contamination) (Khan et al. 2021). C_d_ is evaluated according to the mean concentrations of PTEs for EPA and WHO standards.

The heavy metal pollution index (HPI) is applied to determine total water quality in terms of PTEs. HPI is determined the integrated impact of all PTEs (heavy metals) on water quality. HPI is estimated based on the weighted arithmetic quality average methods (Eq. 3) (Balakrishnan and Ramu 2016; Asim and Nageswara Rao 2021).

3 $$HPI = \frac{\sum_{i=1}^{n}{W}_{i} {Q}_{i}}{\sum_{i=1}^{n}{W}_{i}}$$

*W*_*i*_ and *Q*_*i*_ are the unit weight of individual heavy metal and sub-index of *i*th heavy metal, respectively. *W*_*i*_ is also estimated by Eq. 4 (Asim and Nageswara Rao 2021).

4 $${W}_{i} = \frac{k}{{S}_{i}}$$

where *k* and *S*_*i*_ are proportionality constant and standard value of heavy metal concentration, respectively. It should be stated that *k* = 1 and *W*_*i*_ values are between 0 and 1. Q_i_ is evaluated by Eq. 5 (Mahato et al. 2017).

5 $${Q}_{i} =\left(\frac{{M}_{i} - {I}_{i}}{{S}_{i} - {I}_{i}}\right) \times 100$$

where *M*_*i*_ and *I*_*i*_ are the measured (actual) values of heavy metal concentration (mean concentrations of PTEs) and ideal values of heavy metal concentration, respectively. The values of *I*_*i*_ are depicted in Table 2 (Milivojević et al. 2016; Mahato et al. 2017; Kumar et al. 2020b; Asim and Nageswara Rao 2021; Sheeja and Harilal 2022). The sign (-) demonstrates the numerical differences between two values, neglecting the algebraic sign (Mahato et al. 2014). HPI is categorized into excellent (< 50), good (50–99.99), poor (100–199.99), very poor (200–299.99), and inappropriate for drinking (> 300) (Sheeja and Harilal 2022).

### Exposure and health risk assessment of PTEs in drinking water samples

PTEs can enter the human body through oral intake (ingestion via drinking water), dermal contacts, food chain, and inhalation. Oral intake is more important than others (Jiang et al. 2021; Muhammad et al. 2011). HRA is determined according to the risk level of contaminants and classified as noncarcinogenic and carcinogenic health risks (Kamarehie et al. 2019; Jafari et al. 2021). The present research was conducted to estimate the HRA of PTEs for Shiraz’s children and adult populations through oral intake and ingestion of drinking water. PTE concentrations were applied to evaluate chronic daily intake (CDI) (mg/kg/day) that related to the consumption of water (Eq. 6) (Heshmati et al. 2021; Liu et al. 2022):

6 $$\mathrm{CDI}= \frac{\mathrm{C }\times \mathrm{IR }\times \mathrm{EF }\times \mathrm{ED}}{\mathrm{f }\times \mathrm{ BW }\times \mathrm{AT}}$$

where C, IR, EF, ED, f, BW, and AT represent the contaminant concentration (µg/L), water intake rate (children: 1.25 and adults: 1.95 L/day), exposure frequency (children: min: 180/max: 365/mode: 345 and adults: min: 180/max: 365/mode: 345 Days/year), exposure duration (children: 6 and adults: 50 years), conversion coefficient (1000: µg/L to mg/L), body weight (children: 10.64 and adults: 61.68 kg), and average lifetime (noncarcinogenic: ED × 365 and carcinogenic: 70 × 365 days), respectively (Kumar et al. 2020a, b; Ravindra et al. 2019; Sawut et al. 2018; Wang et al. 2020). In addition, the hazard quotient (HQ) was employed to estimate the noncarcinogenic risk of PTEs exposure in children and adults of Shiraz city (Eq. 7).

7 $$\mathrm{HQ}= \frac{\mathrm{CDI}}{\mathrm{RfD or TDI}}$$

where RfD is reference dose (Mn: 0.14, Hg: 0.0003, and Fe: 0.7 mg/kg/day) (Bortey-Sam et al. 2015; Paul et al. 2019). Tolerable daily intake (TDI) is only applied in HQ formula for Pb (0.0035 mg/kg/day) (Ghoochani et al. 2019). HQ < 1 implies that the contaminant does not have hazardous noncarcinogenic health effects on exposed people, and the noncarcinogenic health risk of contaminants on exposed people is more than the permissible limit for HQ > 1. It means that noncarcinogenic health effects can occur in the future. The greater value of HQ means enhancing the possibility of hazardous noncarcinogenic health effects (Kamarehie et al. 2019; Soleimani et al. 2022).

The integrated risk of PTEs was estimated by hazard index (HI) (Eq. 8).

8 $$\mathrm{HI}= \sum {\mathrm{HQ}}_{\mathrm{i}}={\mathrm{HQ}}_{\mathrm{Hg}} +{\mathrm{HQ}}_{\mathrm{Pb}} + {\mathrm{HQ}}_{\mathrm{Fe}} + {\mathrm{HQ}}_{\mathrm{Mn}}$$

Additionally, carcinogenic risk (CR) of Pb was estimated by Eq. 9.

9 $$\mathrm{CR}=\mathrm{CDI }\times \mathrm{SF}$$

where SF is a carcinogenic slope factor of Pb (0.0085 kg/day/mg) (Nkpaa et al. 2016; Ravindra and Mor 2019). Slope factor (SF) is applied to estimate the carcinogenic risk of contaminants. SF can estimate the probability of upper-bound lifetime of exposed people with cancer diseases over a while. The exposure to contaminant concentrations and the risk of cancer have a linear relationship, and the slope of this relationship is defined as SF. The United States Environmental Protection Agency (USEPA) stated that the CR value between E − 06 and E − 04 is considered a threshold cancer risk. The value < E − 06 can be eliminated for further consideration (carcinogenic risk is negligible) (Nkpaa et al. 2016). However, the value > E − 04 is posed as a potent carcinogen (Jiang et al. 2021).

#### Monte Carlo simulation 

Due to the inadequacy and imprecision of environmental data, numerous factors can be applied in the HRA. HRA contains uncertainty in most cases as sampling errors, measurement errors, and estimates according to judgments. Thus, Monte Carlo simulation (MCS) was suggested by the USEPA, which can overcome the probability and uncertainty of HRA. MCS can quantify the uncertainty in probabilistic frames by computer simulation (Ali et al. 2012; Soleimani et al. 2022) that, in this case, MCS gives better and more reliable exposure assessment and health risk recognition (Ghaderpoori et al. 2020). The application of MCS in environmental risk and public health can cause considerable and vital enhancement in the scientific rigor of these assessments (Qu et al. 2012). C, IR, EF, ED, BW, AT, RfD, and SF distribution factors are log-normal, normal, triangular, fixed, log-normal, fixed, fixed, and fixed, respectively. Also, Oracle Crystal Ball® software (version 11.1.2.3) was employed to run the MCS technique with 10,000 repetitions.

### Statistical analysis

Normality test of data (Kolmogorov–Smirnov), statistical significance at a 95% confidence level (Mann–Whitney and *t*-test), and Spearman rank correlation coefficient (Spearman’s rho) were carried out by the SPSS version. 26. It should be noted that maps were prepared via ArcGIS (version 10.8).

## Results and discussion

### Concentration and spatial distribution of PTEs in drinking water samples

According to the reports, about 96% of Iranian cities have access to safe water supply systems (Alidadi et al. 2019). However, there is still the possibility of contaminating drinking water with PTEs. Nevertheless, factors like source water, pipeline corrosion, inefficient purification system, and dynamics of water molecules can influence the PTEs concentrations in water distribution systems (Alidadi et al. 2019). Table 1 demonstrates the concentrations of Pb, Hg, Mn, and Fe in the drinking water of Shiraz in winter and summer. It should be noted that the mean concentrations of PTEs were compared to the standard values of the Environmental Protection Agency (EPA) and the World Health Organization (WHO). The Kolmogorov–Smirnov test revealed that Hg, Mn, and Fe concentrations were nonnormally distributed in the winter (cold) and summer (warm) seasons. However, the concentration of Pb was normally distributed in the winter and summer seasons. Also, the Mann–Whitney and *t*-test results showed significant differences in Hg, Fe, and Mn concentrations in winter and summer, whereas there was no significant difference in Pb concentrations in winter and summer (at a confidence level of 95%, *p* value = 0.05). It means that variation of seasons had impacts on Hg, Fe, and Mn concentrations. Saeedi et al. (2007) stated that Fe concentration in the warm season was more than in the cold season, the same as in the present study. They also reported that increasing the concentration of Fe in the warm season can be due to decreasing the flow rate of water and the entrance of agricultural runoff to the surface and groundwater resources (containing fertilizers, pesticides, and herbicides). Lower concentrations of Fe in the cold season can be due to increasing water flow rate and the suspended load of water resources (metals like Fe can easily adsorb on the surface of the suspended load). Significant differences between Hg, Fe, and Mn may be due to the different sources of these metals compared to other metals (like Pb) (Astani et al. 2021). Abdollahi et al. (2020) reported that there was no significant difference in the Pb concentration of groundwater samples around landfill (infiltration of landfill leachate into the groundwater resources did not impact Pb concentration). It concluded that variation of seasons did not considerably impact the Pb concentration of Shiraz’s drinking water. Pb has a continuous source that is not different in the warm and cold seasons. In addition, the results showed that mean concentrations of Pb, Hg, Mn, and Fe were lower than EPA and WHO standards in all water samples.

**Table 1:** PTE concentrations and degree of contamination in the drinking water of Shiraz

PTEs	Range (µg/L)		Mean (µg/L) (*M*_*i*_) ± SD		S_i_ (µg/L)		Cf_i_			
	W	S	W	S	EPA	WHO	EPA		WHO	
							W	S	W	S
Pb	0.22–0.60	0.11–3.80	0.36 ± 0.08	0.50 ± 0.62	15	10	− 0.98	− 0.97	− 0.96	− 0.95
Hg	0.17–0.60	0.10–0.60	0.32 ± 0.12	0.20 ± 0.11	2	6	− 0.84	− 0.90	− 0.95	− 0.97
Mn	0.28–16.23	0.19–3.68	2.28 ± 3.91	0.55 ± 0.57	50	400	− 0.95	− 0.99	− 0.99	− 0.99
Fe	3.69–32.53	6.86–26.64	8.72 ± 6.35	10.36 ± 3.04	300	300	− 0.97	− 0.96	− 0.97	− 0.96
${\mathrm{C}}_{\mathrm{d}}=\sum {\mathrm{Cf}}_{\mathrm{i}}$							− 3.74	− 3.82	− 3.87	− 3.87

*SD* standard deviation, *W* winter, *S* summer

**Fig. 2:**
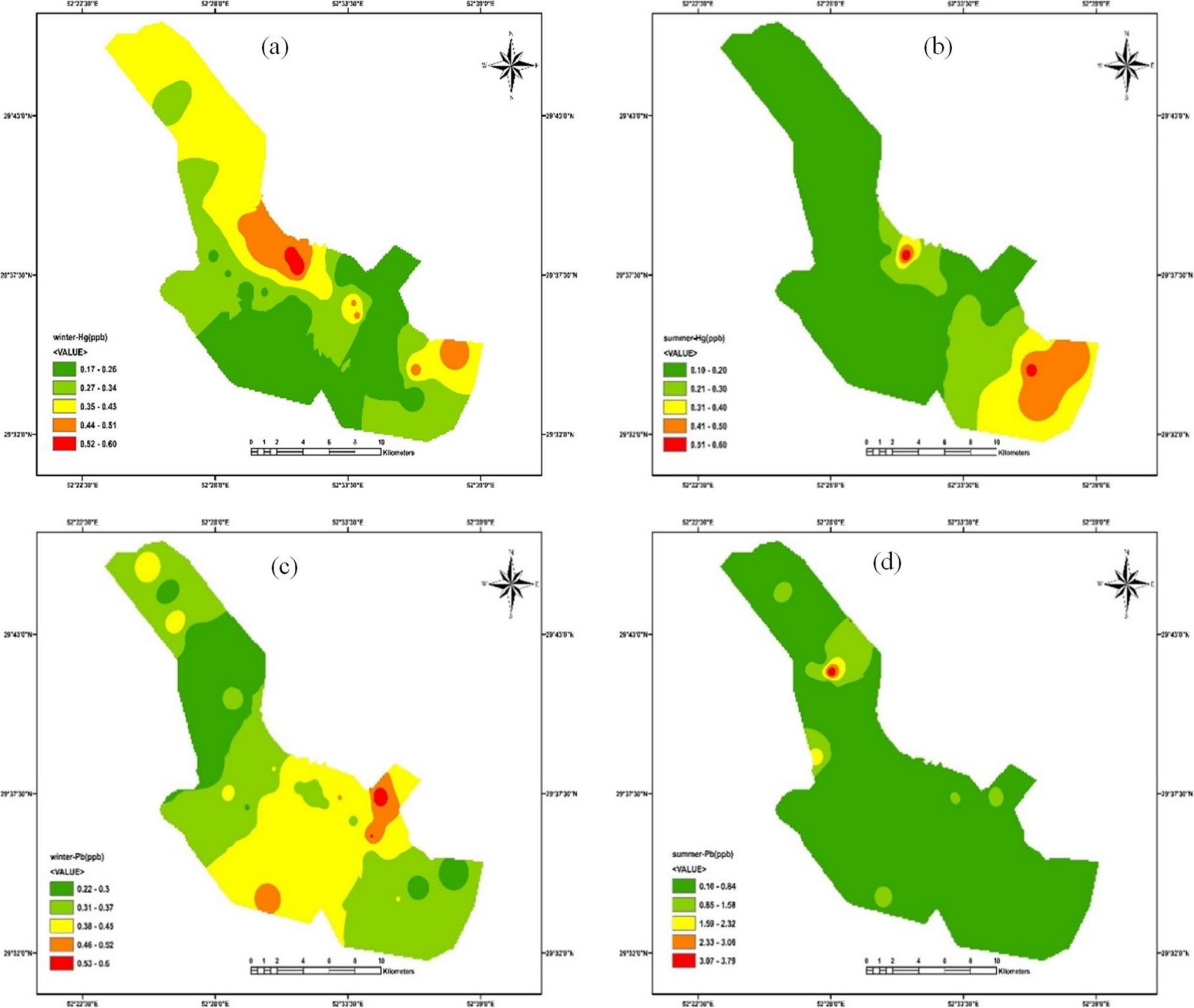

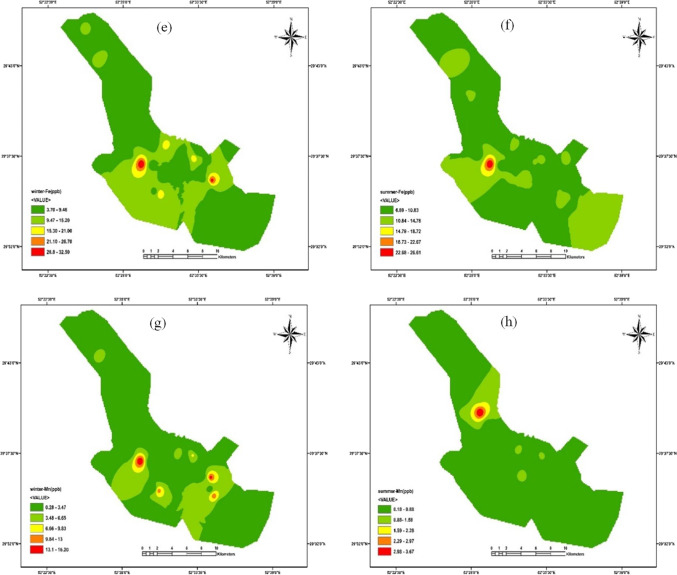
The distribution of HM concentrations in drinking water of Shiraz between winter and summer (**a** Hg concentrations in winter, **b** Hg concentrations in summer, **c** Pb concentrations in winter, **d** Pb concentrations in summer, **e** Fe concentrations in winter, **f** Fe concentrations in summer, **g** Mn concentrations in winter, and **h** Mn concentrations in summer)

The distribution of PTEs concentrations is shown in Fig. 2. The most important reasons for the existence of Pb in drinking water may be due to direct leaching of Pb from pipes, the disintegration of brass fittings, and detachment of old solder. Lead pipes are considered a consistent source of Pb in plumbing systems even after years of installation (Wang et al. 2014). It should be noticed that copper pipes contain impurities like Pb. Pb is commonly used to construct copper and copper-alloy tubes and fittings to enhance machinability. In this case, Pb can enter pipeline systems' drinking water and affect water quality (Lee et al. 2016). The mean concentration of Pb in winter was lower than in summer.

Goovaerts (2017) reported that Pb concentration in cold months is lower because Pb cannot easily be dissolved in chilled water. Polymer-based material pipes like polyvinyl chloride (PVC) have been substituted to lead pipes these days. Stabilizers like Pb, Cd, Ca, and Ba are consumed in PVC pipes production. The PVC pipes contain 1.8% wt Pb in their structure as lead phosphite, lead sulfate, lead stearate, and lead phthalate. Thus, applying PVC pipelines in plumbing systems leads to the entrance of Pb into the drinking water. Al-Malack (2001) reported that PVC pipelines with lead stabilizers leach about 1000 µg/L of Pb to water within 48 h at pH 5, and the concentrations of other stabilizers (Cd, Ca, and Ba) were also increased in PVC pipelines by increasing the time (Al-Malack 2001; Harvey et al. 2015; Lasheen et al. 2008).

The precise reasons for the presence of Hg in drinking water are still controversial. Based on the results of different investigations, low organic carbon content and high salinity (especially the presence of chloride) in the sediments of the aquifer lead to the desorption and transportation of Hg to the aquatic phase. Organic compounds have affinity and complexing capacity to Hg. When the chloride (Cl) concentration increases, Hg desorbs from the metal hydroxide compounds, which leads to the generation of the Hg-Cl soluble compound (Bone et al. 2007; Szymczycha et al. 2013). However, this research did not examine the effective parameters for Hg concentration.

The mean concentration of Fe was higher than the other PTEs in winter and summer. The application of galvanized pipelines, steel pipelines, ductile cast iron pipelines, and grey cast iron pipelines are other reasons for the existence of Fe in potable water. Fe is consumed in the structure of the mentioned pipelines (Liu et al. 2016). The corrosion of iron products and subsequently release of Fe to drinking water can supply favorable conditions for the growth of bacteria (formation of suspended and biofilm of bacteria). Bacterial cells can easily accumulate on the iron corroded structure and iron minerals in drinking water plumbing systems. The existence of charges on the surface and the high surface area of iron oxides may enhance the attachment and colonization of microorganisms that lead to the clogging and reduction of water flow in the pipelines.

The application of iron-based coagulants and their remaining is another factor for the existence of Fe in drinking water. Fe also affects water’s visual characteristics and taste (Chaturvedi and Dave 2012). In addition, the Fe concentration in summer was higher than in winter, possibly due to water temperature. According to the Arrhenius-type equation, microbial strains’ growth and activity are increased by increasing the temperature (limited range of temperature). Thus, in this case, increasing the biological activity may cause more corrosion and release elements like Fe into the water. The variation in the temperature range (between winter and summer) can cause mechanical stresses, which lead to the formation of cracks or spalling, and the release of metals into the water pipelines (McNeill and Edwards 2002; Teng et al. 2008).

Insufficient Mn removal at the water treatment plants leads to the entrance of soluble Mn into the water distribution systems (Cerrato et al. 2006). Manganese bacteria grow on the walls of pipelines in the existence of Mn in water, and the corrosion of pipeline systems can occur in this condition (Okoniewska et al. 2007). The undesirable effects of Mn in drinking water are related to aesthetic problems (Cerrato et al. 2006). It should be noted that the concentration of PTEs obeyed the following sequence: Fe > Mn > Pb > Hg in the present study, which is consistent with the Liu et al. (2016) study. They also reported that the concentration of PTEs obeyed the following sequence: Fe > Mn/Al/Zn > Pb/Cu > Cr > Cd in the drinking water of China (Zhejiang Province).

**Fig. 3:**
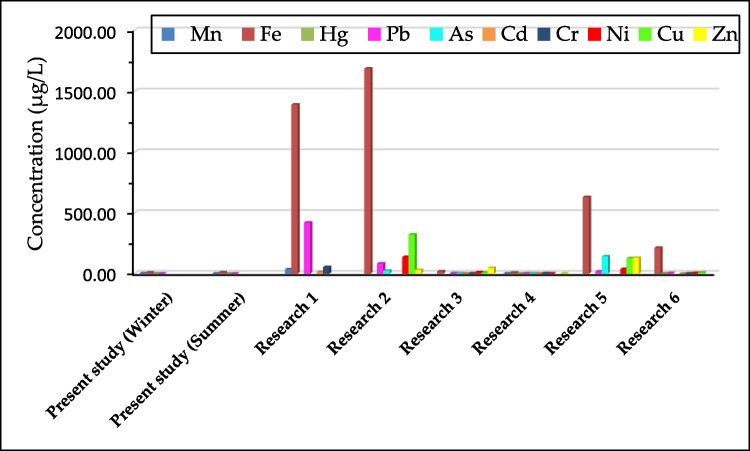
Concentration of PTEs in groundwater resources (wells) of Shiraz and the present research

Water resources and their quality are the other influential practical factors in the existence of pollutants in drinking water. The drinking water of Shiraz is supplied from the Doroudzan dam (on the Kor River) and wells (groundwater) (“abfa-shiraz.ir” Abfa-shiraz.ir 2014), which different chemical substances may pollute. Ebrahimi and Taherianfard (2011) investigated the PTEs concentrations in the water and fish of the three sites of the Kor River (Doroudzan dam as the upper sampling zone, middle sampling zone, and lower sampling zone). They reported that the PTEs (As, Pb, Hg, and Cd) were present in all water and fish samples from different sampling zones of the Kor River. They also reported that PTEs lead to the disruption of reproductive hormone secretion and inducement of pathological changes in fishes. The mean concentrations of PTEs in the present research were compared with Shiraz’s other groundwater resources (wells). As depicted in Fig. 3, PTEs were present in all wells consumed as drinking water, even in lower and higher concentrations than in the present research (Shakeri et al. 2009; Amin et al. 2011; Godarzi and Samani 2012; Karami et al. 2016; Madadi and Madadi 2019; Fattahzadeh et al. 2021). Based on the results of Table 1, Cf_i_ and C_d_ values for Pb, Hg, Mn, and Fe in winter and summer were less than one. It means that the contamination degree of Shiraz’s drinking water is significantly low (Khan et al. 2021). Table 2 proves that HPIs in winter and summer (for both EPA and WHO standards) were less than 50 and categorized into the excellent group (Sheeja and Harilal 2022).

**Table 2:** Heavy metal pollution index of PTEs in drinking water of Shiraz

PTEs	*M*_*i*_ (µg/L)		*S*_*i*_ (µg/L)		*I*_*i*_ (µg/L)	*W*_*i*_ (µg/L)		Q_i_				W_i_Q_i_			
	W	S	EPA	WHO		EPA	WHO	EPA		WHO		EPA		WHO	
								W	S	W	S	W	S	W	S
Pb	0.36	0.50	15	10	-	0.0666	0.1000	2.40	3.33	3.60	5.00	0.16	0.22	0.36	0.50
Hg	0.32	0.20	2	6	-	0.50000	0.1666	16.00	10.00	5.33	3.33	8	5	0.89	0.55
Mn	2.28	0.55	50	400	100	0.0200	0.0025	195.44	198.90	32.57	33.15	3.91	3.98	0.08	0.07
Fe	8.72	10.36	300	300	-	0.0033	0.0033	2.91	3.45	2.91	3.45	0.01	0.01	0.01	0.01
						$\sum {W}_{i}$						$\sum {W}_{i} {Q}_{i}$			
						0.58	0.27					12.08	9.21	1.34	1.13
HPI												20.82	15.88	4.96	4.18

*-*: zero value

Based on the mentioned subjects, the pollutants can quickly transfer from water resources to the water distribution systems due to different parameters, like inadequate treatment in water treatment plants. Also, the corrosion of water supply systems may result in the transfer of contaminants from plumbing systems to the drinking water that can cause probable adverse effects on the potable water of Shiraz (the fifth populated city of Iran). It should be noted that the PTE concentrations were lower than typical values in all samples in the two seasons. However, due to the adverse effects of PTEs on human health, liable institutions should perform continuous and regular considerations on the drinking water of Shiraz.

### Spearman's rho correlation coefficient

Spearman’s rho correlation coefficients of PTEs concentrations were analyzed, and the results are presented in Table 3. The correlation coefficient is categorized to weak (*r* < 0.5), moderate (0.5 < *r* < 0.7), and strong (*r* > 0.7) (Egbueri and Unigwe 2020). Thus, Fe–Mn and Fe-Pb (in winter), Fe–Mn and Fe-Hg (in summer), and Fe-Hg and Pb-Hg (in winter) were grouped in the moderate, weak, and weak categories, respectively. Fe showed a positively significant correlation with Pb (winter). Fe and Pb had the greatest significant association (0.690) in winter, which can be due to the release of Fe and Pb from the same source (pipelines) (Jakhu and Mehra 2018). Hg showed negatively significant correlations with Fe (− 0.322) and Pb (− 0.436) (winter). The negative correlation of Hg with other elements was reported in the other studies. Alinejad et al. (2016) stated that Hg showed a negative correlation coefficient with Fe in the drinking water resources of Kohgiluyeh county. The absence of correlations between PTEs means that the concentrations of PTEs were not controlled by a single parameter (Hussain et al. 2019). Mn showed significant correlations with Fe in winter (0.619) and summer (0.340). It can be due to the redox cycling of Mn, which can control Fe concentrations (Satheeshkumar and Senthilkumar 2011).

**Table 3:** Spearman’s rho correlation coefficient between PTEs in winter and summer

		Mn	Fe	Hg	Pb
Winter	Mn	1.000			
	Fe	0.619**	1.000		
	Hg	− 0.070	− 0.322*	1.000	
	Pb	0.286	0.690**	− 0.436**	1.000
Summer	Mn	1.000			
	Fe	0.340*	1.000		
	Hg	0.065	0.376*	1.000	
	Pb	− 0.051	0.022	− 0.286	1.000

^*^Significance at the 0.05 level (2-tailed)

^**^Significance at the 0.01 level (2-tailed)

### Health risk assessment

**Fig. 4:**
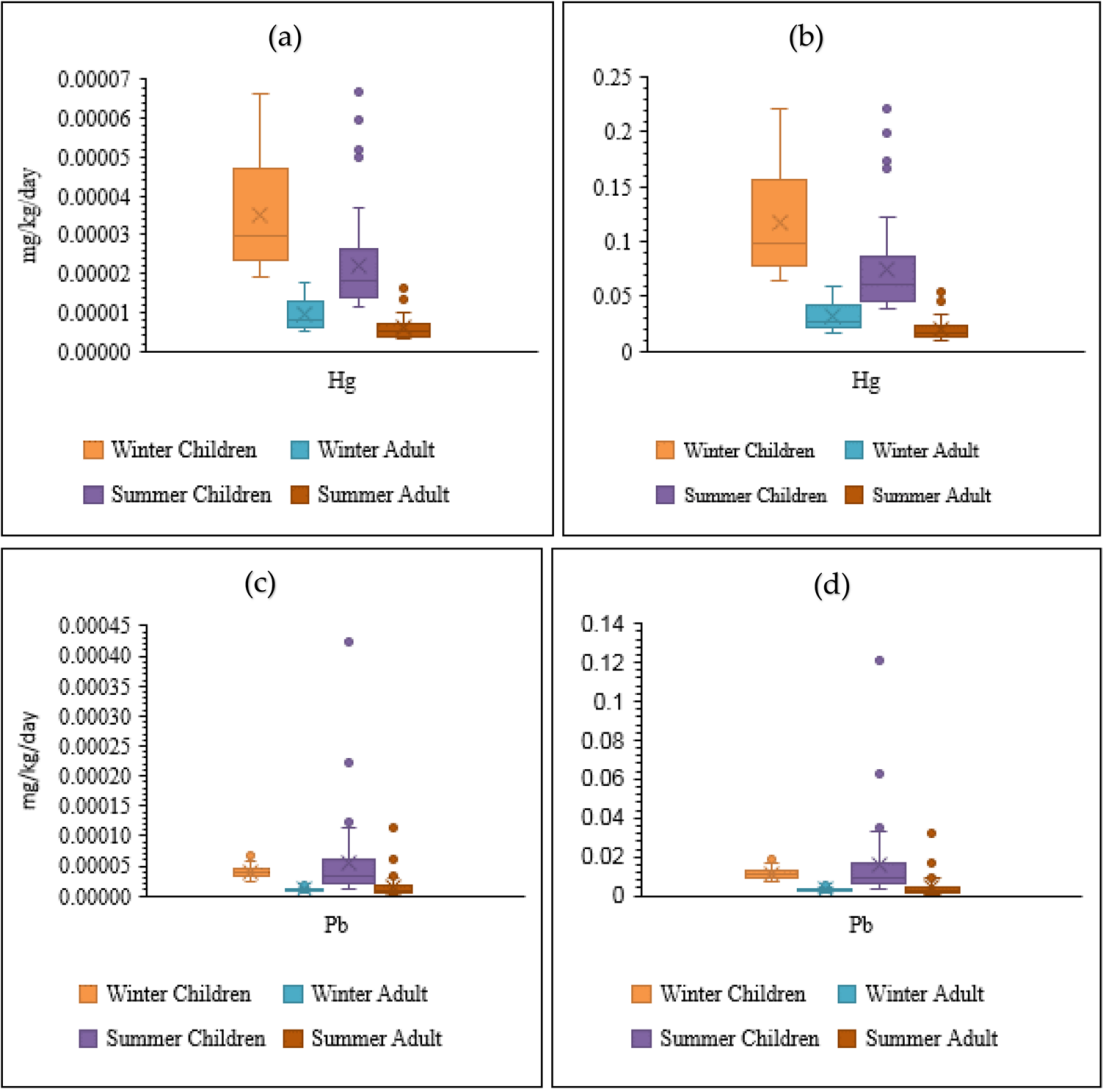

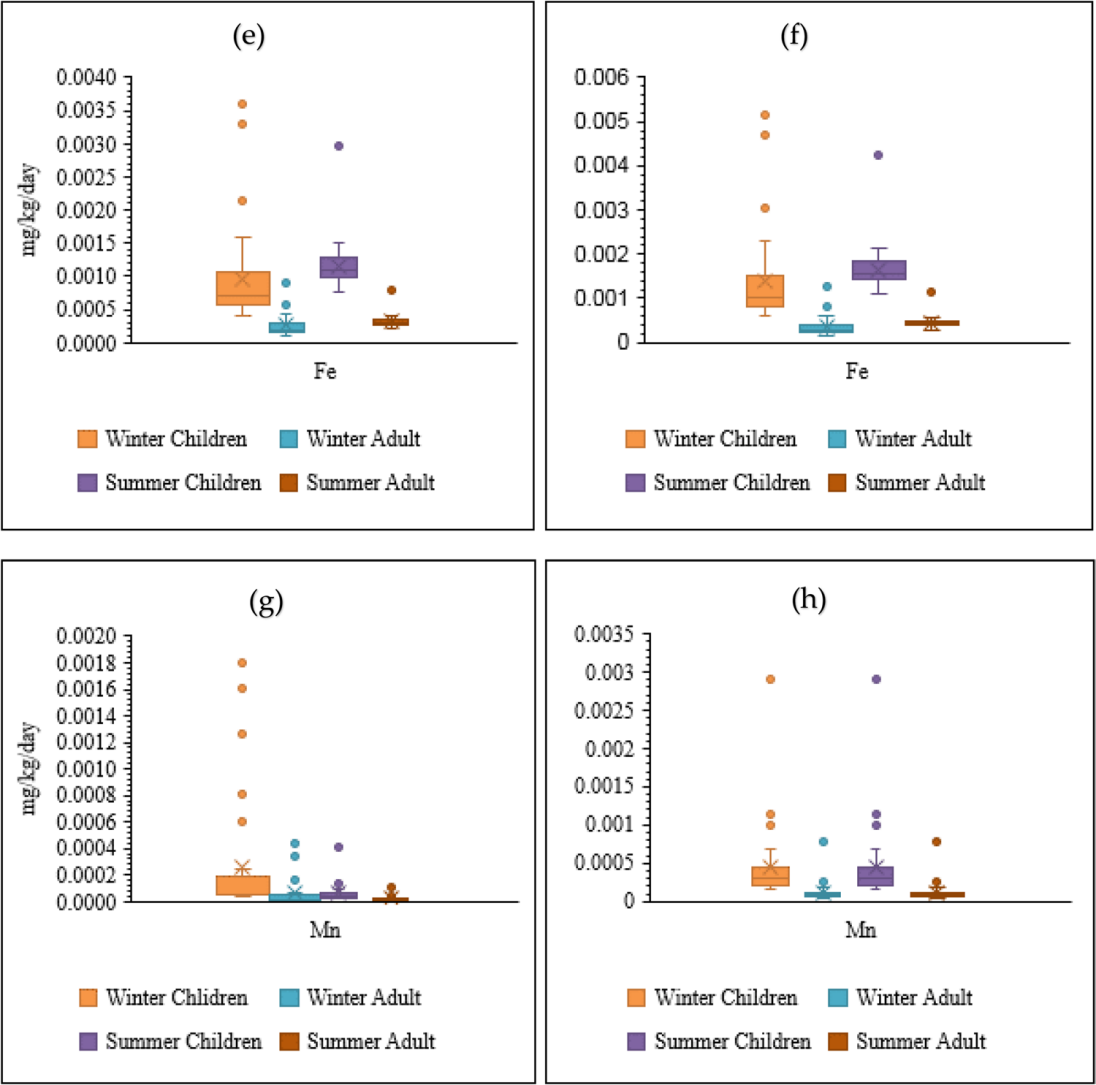
CDI and noncarcinogenic health risks (HQ) posed by HMs in drinking water of Shiraz through ingestion (**a** CDI of Hg; **b** HQ of Hg; **c** CDI of Pb; **d** HQ of Pb; **e** CDI of Fe; **f** HQ of Fe; **g** CDI of Mn; **h** HQ of Mn)

HRA is an effective technique in determining the importance and nature of detrimental health impacts in humans exposed to poisonous compounds in contaminated environments. Humans can be exposed to PTEs through drinking water, inhaled aerosol (dust and particles), and food (Mohammadi et al. 2019). Daily intake is the most important factor in the toxicity of PTEs in humans. Thus, this study investigated daily intake through drinking water ingestion. Figure 4 depicts that CDIs (mg/kg/day) (mean values) of PTEs were 0.00025 (winter, children), 0.00007 (winter, adult), 0.00006 (summer, children), and 0.00002 (summer, adult) for Mn, 0.0010 (winter, children), 0.0003 (winter, adult), 0.0012 (summer, children), and 0.0003 (summer, adult) for Fe, 0.00004 (winter, children), 0.00001 (winter, adult), 0.00005 (summer, children), and 0.00001 (summer, adult) for Pb, and 0.00003 (winter, children), 0.00001 (winter, adult), 0.00002 (summer, children), and 0.000005 (summer, adult) for Hg. CDIs followed the sequence Fe (summer, children) > Fe (winter, children) > Fe (summer, adult) > Fe (winter, adult) > Mn (winter, children) > Mn (winter, adult) > Mn (summer, children) > Hg (summer, adult) > Pb (summer, children) > Pb (winter, children) > Hg (winter, children) > Hg (summer, children) > Mn (summer, adult) > Pb (summer, adult) > Pb (winter, adult) > Hg (winter, adult). As shown in Fig. 4, all the PTEs had HQs < 1. The health risk consideration proved an acceptable level of noncarcinogenic harmful risk of Pb, Hg, Mn, and Fe in the drinking water of Shiraz. HQs followed the sequence Hg > Pb > Mn > Fe and Hg > Pb > Fe > Mn for children and adults in winter and summer, respectively. As presented, the highest HQs were observed for Pb and Hg.

**Fig. 5:**
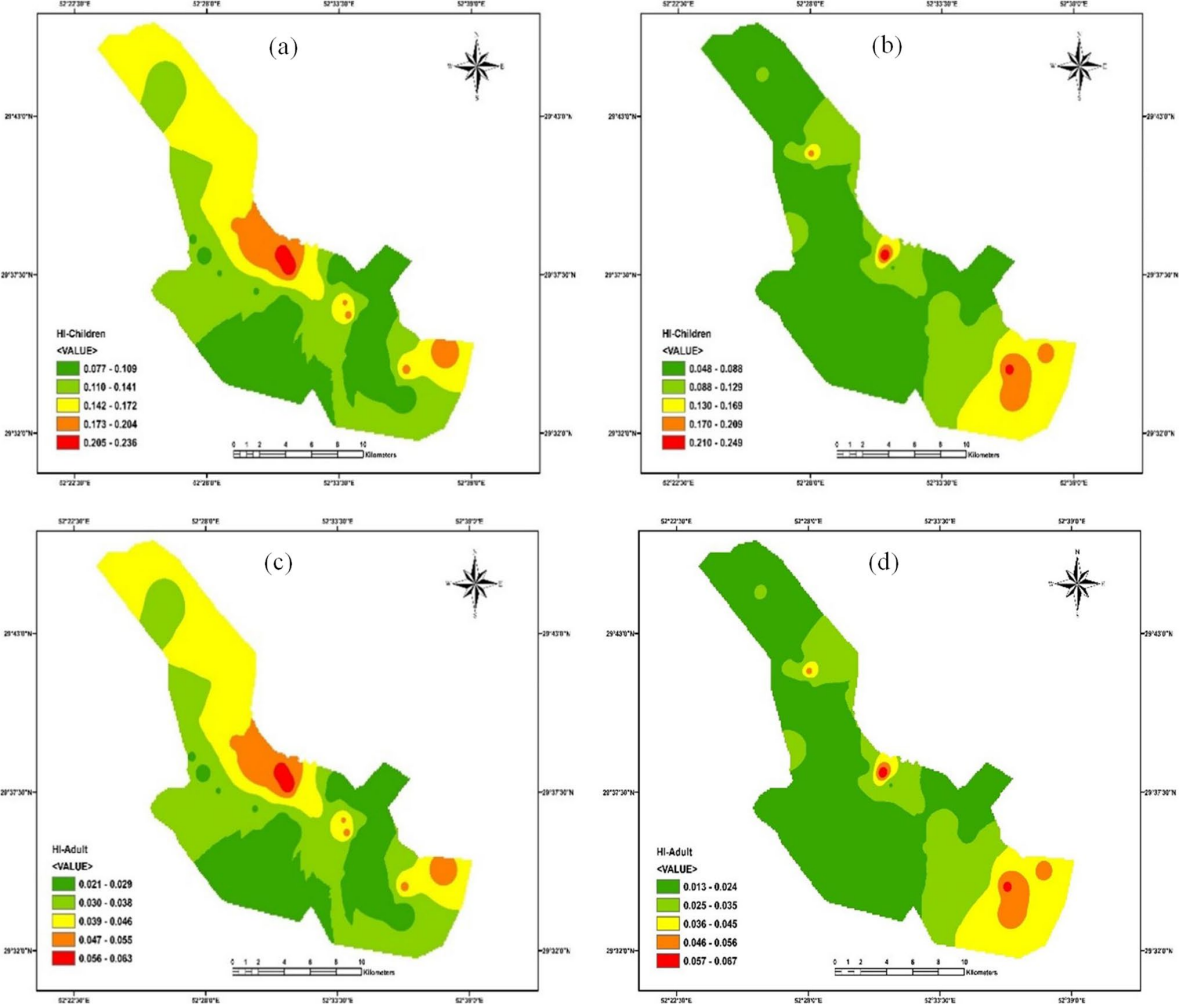
HI, distribution (**a** children, winter; **b** children, summer; **c** adults, winter; **d** adults, summer)

It means that the possibility of noncarcinogenic hazardous effects may increase for Hg and Pb in children and adults. Based on the results, higher CDIs and HQs were observed for children in winter and summer for all PTEs. It has been proved that children are confronted with the highest noncarcinogenic risk and are more susceptible to contaminants (because of their low body weight). As shown in Fig. 5, HIs were less than one and were safe for human health. Also, children’s HIs were higher than adults in winter and summer. Children are more susceptible to contaminants’ chronic, subacute, and acute influences (Bortey-Sam et al. 2015). HIs > 1 demonstrate the potential of adverse human health effects and the requirement for further investigation (Golaki et al. 2022; Şener et al. 2017). It should be stated that the summation of HIs (summation of winter and summer means) for children (0.22) and adults (0.06) was less than one in the present research.

HRA of PTE exposure via drinking water was investigated in different cities of Iran. PTEs (Cr, Pb, and Cd) in groundwater resources of Torbat-e Heydariyeh city in south-eastern Iran had HI > 1 for infants (Soleimani et al. 2022). Fallahzadeh et al. (2017) stated that the drinking water resources of five cities in Yazd Province (central part of Iran) had HQ < 1 for Cu, Fe, Zn, Cr, Mn, Pb, and Ni. Mohammadi et al. (2019) reported that the drinking water of Khorramabad (west of Iran) had HQ and HI less than one (Ba, Pb, Mo, Cr, Ni, Cd, Zn, and Cu). Compared with the other studies of HRA in different cities of Iran, Shiraz’s drinking water is safe for oral consumption for children and adults. However, continuous oral drinking water containing PTEs can enhance CR in children and adults. CR values were in the range of 1.78E − 08 to 4.85E − 08 (mean = 2.88E − 08) and 3.99E − 08 to 1.08E − 07 (mean = 6.46E − 08) for children and adults in winter, respectively. CR was 2.88 in 100,000,000 for children, while CR was 6.46 in 100,000,000 for adults. Also, CR values were in the range of 8.56E − 09 to 3.08E − 07 (mean = 4.03E − 08) and 1.92E − 08 to 6.90E − 07 (mean = 9.04E − 08) for children and adults in summer, respectively.

**Fig. 6:**
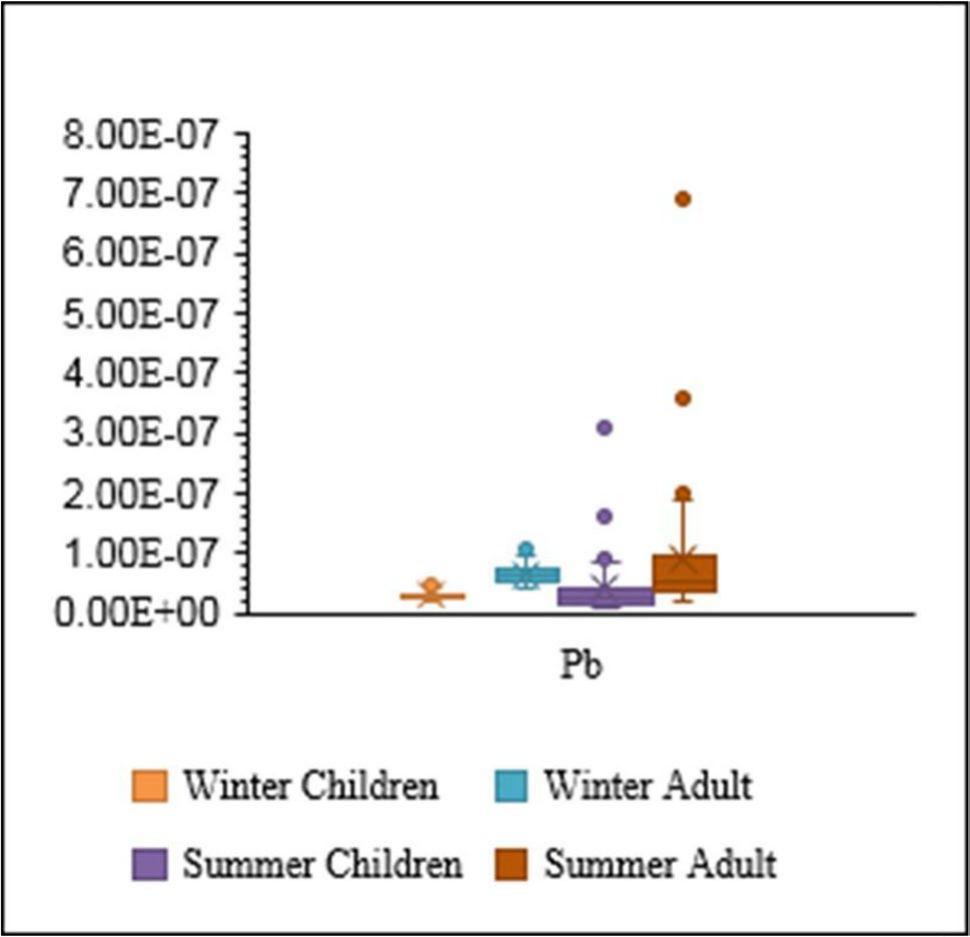
Carcinogenic health risks (CR) posed by Pb in drinking water of Shiraz through ingestion

According to the observations, all CR values were less than E-06 for Pb in winter and summer (Fig. 6). The summation of CRs (summation of winter and summer means) was less than E − 06. Thus, CR can be neglected and eliminated (Nour et al. 2022). In general, the results of C_d_ and HPI indicated that Shiraz’s drinking water is healthy and safe for consumers, and remarkable noncarcinogenic (HQ and HI) and carcinogenic (CR) impacts of PTEs (Pb, Hg, Mn, and Fe) were not observed in the drinking water of Shiraz. Due to the feasibility of unforeseeable contaminations in the future, the general evaluation is urgent. In this case, old water pipes should be replaced by new ones in the transportation and distribution systems. Due to water scarcity, new resources may be applied for drinking water. Thus, the concentrations of PTEs in new water resources should be carefully controlled. Also, efficient treatment should be applied in the water treatment plant of Shiraz now and in the future.

### Monte Carlo simulation

**Fig. 7:**
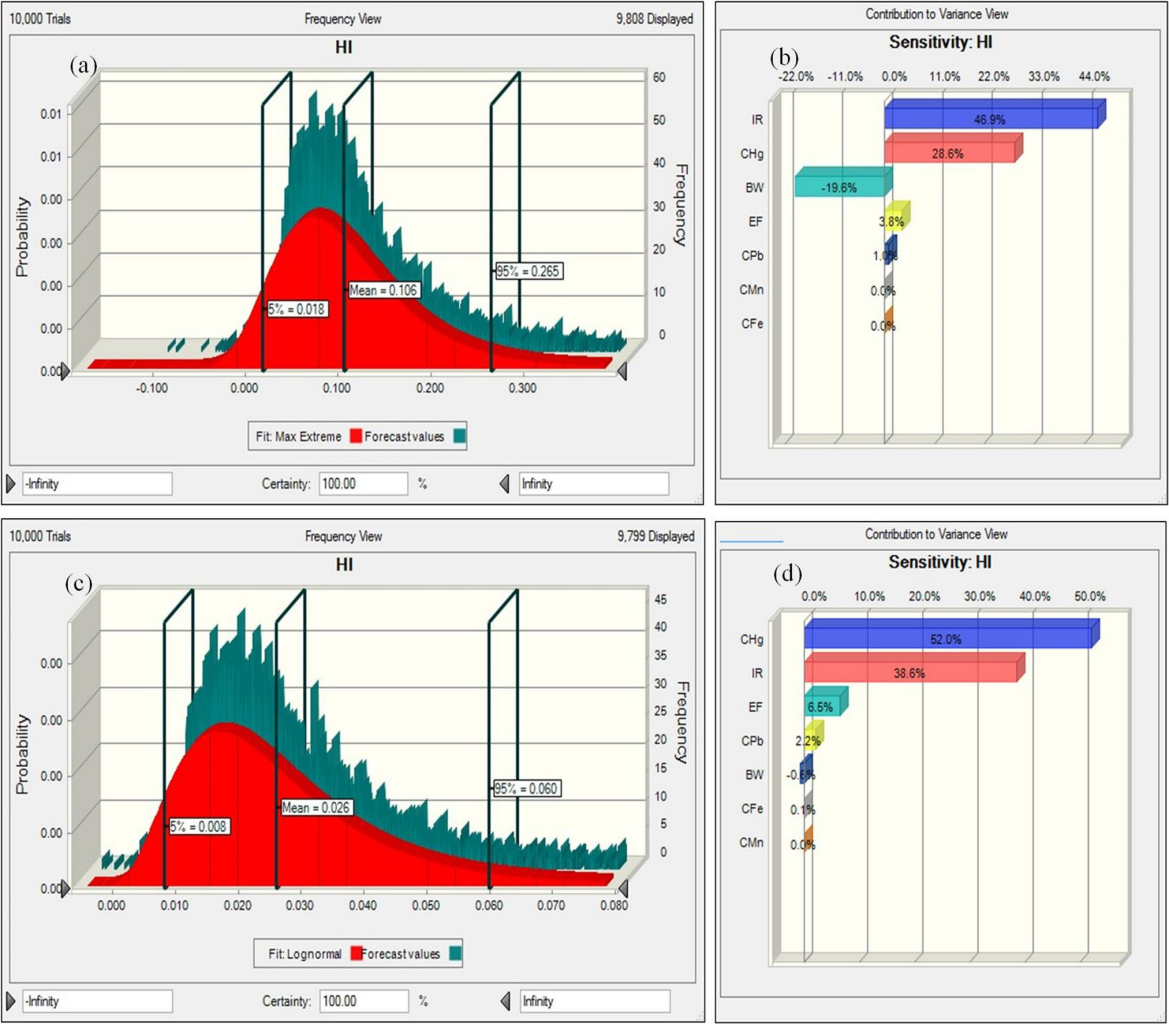

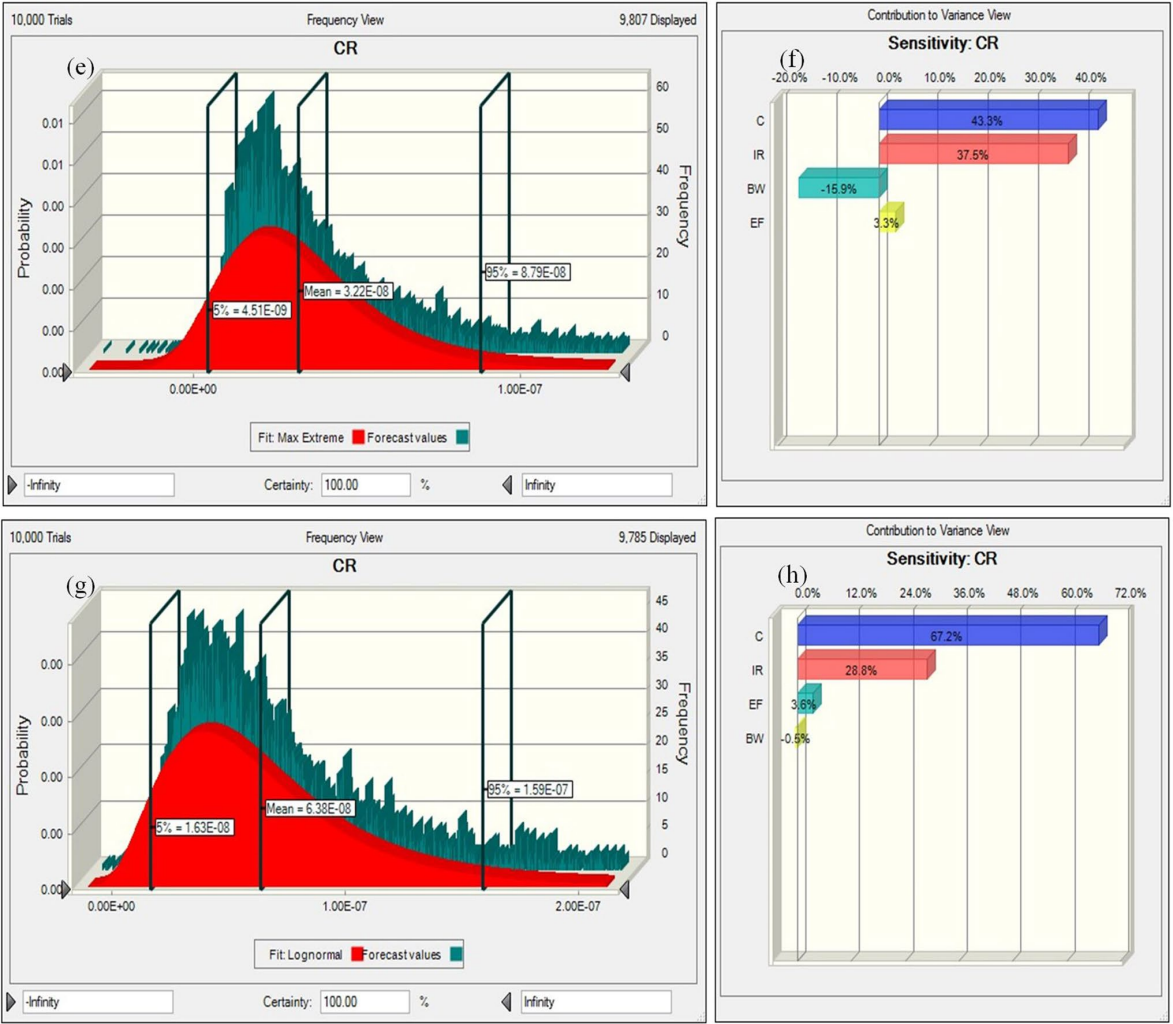
Histograms of the uncertainty analysis of HMs (HI and CR) and the contribution of the input variable to the uncertainty of estimated HI and CR (**a** histogram of HI for children, **b** sensitivity of HI for children, **c** histogram of HI for adults, **d** sensitivity of HI for adults, **e** histogram of CR for children, **f** sensitivity of CR for children, **g** histogram of CR for adults, **h** sensitivity of CR for adults)

As described, HI was estimated by Eq. 8, and MCS also estimated the variance of HI. The probabilistic approaches for PTEs in the exposed groups (children and adults) were performed by considering the appropriate distribution of effective parameters (ingestion rate, concentration of contaminant, exposure frequency, and body weight), and the histogram plots have been shown in Fig. 7. As shown in Fig. 7 (histogram a and c), the probability estimation proved that the HI level in children (mean = 0.106) was more than in adults (mean = 0.026). Additionally, the values of 0.018–0.265 and 0.008–0.060 were observed for the 5th and 95th percentile in children and adults, respectively.

The results proved that HI values in children and adults were less than one. It means that the probability of noncarcinogenic risks and detrimental impacts of water intake is not enhanced with long-term exposure to PTEs (Pb, Hg, Mn, and Fe) through drinking water ingestion. Histograms e and g present Pb’s carcinogenic risk assessment values at the 5th and 95th percentile confidence levels for children and adults. The CR value of Pb in adults (mean = 6.38E − 08) was more than in children (mean = 3.22E − 08). It demonstrates that Pb had the greatest risk for adults. Also, the most efficient factor in enhancing the health risk in exposed groups (children and adults) was determined by sensitivity analysis. Sensitivity analysis can characterize the factors that influence risk assessment most (Mohammadpour et al. 2022a, b). In this approach, the final output's uncertainty is influenced by input factors’ variability (Bazeli et al. 2022).

Figure 7b and d present the sensitivity analysis of effective factors in estimating HI for exposed children and adults. Based on the observations, the water intake rate greatly enhanced the noncarcinogenic risk for children. However, Hg concentration had the greatest impact on enhancing the noncarcinogenic risk for adults, and a decline in Hg can decrease the risk of health. Thus, it is suggested that appropriate strategies should be taken to minimize Hg concentration in the potable water of Shiraz.

As presented in Fig. 7b and d, BW had a negative value in sensitivity analysis for children and adults. It demonstrates that BW is inversely related to HI. It means that when BW enhances, HI declines. It should be pointed out that the sensitivity analysis of effective factors in determining CR for exposed children and adults is presented in Fig. 7f and h. According to the observations, the concentration of Pb had the greatest impact on CR in children (43.30%) and adults (67.20%). Also, the results exhibited that BW had negative values for children and adults. The present investigation gives valuable information about the contamination of PTEs and their effect on human health in drinking water. Also, the results can be effective in the application and performance of protective procedures for exposed occupants.

## Conclusion

This research was carried out to determine PTE concentrations in the drinking water of Shiraz. The results proved that Mn and Fe concentrations were more than the other PTEs, which can be due to the characteristics of the pipeline distribution system. Also, the concentrations of Fe and Pb in summer were more than in winter. Correlation analysis presented that PTEs had positive and negative correlations with each other. HRA results proved that HI values for PTEs were below the safe level (< 1). CR of Pb was also negligible. Based on the results of MCS analysis, water intake rate and Hg concentration were the most influential factors in HI for children and adults, respectively. Bodyweight was negatively correlated with HI in children and adults. Pb concentration had the most significant impact on CR in children and adults.

## Data Availability

Not applicable.
